# The Wise Men of Texas

**Published:** 1899-07

**Authors:** 


### The Wise Men of Texas.

For bungling, and the incorrect use of the English language and a general Sairy Gamp-ishness of expression, I will stake an average Texas "statesman" against the civilized world. The Texas legislators remind me of the man who in digging a ditch, threw the dirt in front of him, in such a way that he had always to remove it before he could dig any more ditch. At one session they spend much time in undoing what was done at a former session. That laws should state specifically and unequivocally what is meant has been made manifest in many instances, as the meaning may be greatly changed or obscured, even by the insertion or omission of so small a thing as a comma. Take this illustration: The proprietor of a nostrum sued a newspaper for libel for printing the following testimonial: "After being brought to death's door by the use of two bottles of your Be-ruin-you, I am restored to perfect health." The "testimonial" as furnished to the paper had a comma after "door." By the omission of this comma the meaning of the testimonial was reversed, and the publication of it in that shape constituted a burlesque, if not a libel. The Texas legislature some ten years ago established and endowed a medical college. Meantime to dissect a human body is a felony, and no attempt was ever made to legalize dissection until the immortal Twenty-sixth legislature tried its "prentice hand" on it. That they made a botch of it, and the Governor vetoed it, will be seen from the following veto message of His Excellency. With the aid of a few bracket helps, which we have ventured to insert, this message will, we hope, be intelligible:

THE "HUMANE BILL": VETO.

"AUSTIN, TEXAS, June 15.—To the Secretary of State: I herewith transmit to you without my approval Senate bill No. 81, entitled 'An Act for the promotion of medical science by [the] distribution and use of unclaimed human bodies for scientific purposes [,] through a board created for that purpose [;] and to prevent unauthorized uses [of] and traffic in human bodies [,] and to legalize dissection by authorized persons.'

"My disapproval of the bill rests upon the ground that [,] under its provisions it is made the duty, under penalty [for failure to so do,] of a fine of not less than $100 and not more than $500 [,—] of the superintendents of the eleemosynary and penal institutions of the State, and also [of] all officials having charge of alms houses, prisons, morgues and hospitals, to inform the anatomical board of the death of any inmate [of either of said institutions,] within twenty-four hours thereafter, and to deliver to the board or its agents the body of such deceased person, unless such body be claimed by a relative or friend for burial [;] or unless such deceased person shall, while living, [have] expressed the desire that his [or her] body shall be buried. No notification, however, of the death of such inmate is required to be given to his [or her] friends or kindred, nor is a complete description of the body required to be recorded at the place where it is dissected.

"The measure, in my judgment, affords no protection whatever to a very large class of unfortunate people, and in [under?] its operations the grossest and most scandalous abuses may arise, and that, too, without any violation of the letter of the law.

(Signed) "JOSEPH D. SAYERS,
"Governor."

The apology for a "medical practice act" now on our statute books, in its opening clause is worded as follows: "Any person who shall hereafter practice for pay in any of its branches, medicine," etc.

Texas, by popular vote, agreed to amend the Constitution so that the legislature could pension indigent Confederate soldiers. The immortal Twenty-sixth undertook the job. They botched it, of course. The bill which was enacted, and which His Excellency did *not* veto, provides in one clause that the applicant for pension shall own no property, and in the next clause he is required to file an inventory of his property.

The papers are "jollying" the senator from Harris county. The one single measure which he succeeded in getting through has been knocked out because it is made "retro-active," and is "unconstitutional." His Excellency did not veto it, however; which fact gives the senator a salve for his sore, and the laugh on the other fellow.

The "anti-mob" law has just recently been knocked out also, by a decision from the Court of Criminal Appeals, with the statement that "it is inoperative and void, not being *susceptible of construction*." [!!]

But, what else is to be expected? The great State of Texas pays her "wise men" the munificent sum of two dollars a day after the first sixty days. They are supposed to get rich enough on the five dollars a day which they get for sixty days to stand it; to pay board, etc., and to "set 'em up" for all who have favored their pet measures. You can't make a silk purse out of a sow's ear,—or, as Love puts it: You can't make a milk punch out of a cow's ear. The following "size up" I beg to reproduce here from a former article:

"In past ages, in all ancient nations, even amongst savage tribes, the making of the laws has been entrusted to the wise men. In our day and generation it is left to a heterogeneous body (not class) of men, thrown haphazard together every two years by popular choice, and chosen for any other consideration too frequently, than that of wisdom or fitness, young men predominating as a rule. Young and old and middle-aged, black and white, learned and unlearned, farmer, mechanic, lawyer, merchant, Christian, Jew, Gentile and pagan—hardly any two of them of like habits of thought or educated in the same direction or to the same degree; men who are wise only in their own conceit, too often, and who are notoriously adverse to being advised. The young American statesman as we see him in legislative halls of some states, is a finished product, whom the wisest in this body can not enlighten on any subject, either of science, politics or state government; he knows it all. Nevertheless, the problem of prison reform [or any other reform] can never be solved practically without the co-operation of state legislatures."*

It is hardly necessary to say that there are notable exceptions. In every Texas legislature there are good earnest well-meaning and highly intelligent men, but they constitute such a small minority that their efforts are paralyzed by the avalanche of stupidity, ignorance and self-conceit that is precipitated upon every sensible measure proposed.

*Address before the National Prison Association, December 2, 1897.

